# Molecular cytogenetic analysis of Xq critical regions in premature ovarian failure

**DOI:** 10.1186/1755-8166-6-62

**Published:** 2013-12-20

**Authors:** Artur Beke, Henriett Piko, Iren Haltrich, Judit Csomor, Andras Matolcsy, György Fekete, Janos Rigo, Veronika Karcagi

**Affiliations:** 1 1st Department of Obstetrics and Gynecology, Semmelweis University, Baross u. 27, 1088 Budapest, Hungary; 2 Department of Molecular Genetics and Diagnostics, National Institute of Enviromental Health, Gyáli út 2-6, H-1096 Budapest, Hungary; 3 2nd Department of Pediatrics, Semmelweis University, Üllői út 26, 1085 Budapest, Hungary; 4 1st Department of Pathology and Cancer Research, Semmelweis University, Üllői út 26, 1085 Budapest, Hungary

**Keywords:** Sterility, Premature premature ovarian failure (POF), Primary ovarian insufficiency (POI), FMR1 gene analysis, Array–comparative genomic hybridization (aCGH), X chromosome deletion, Repeat primed PCR, G-banding, Deletion breakpoint, Turner syndrome

## Abstract

**Background:**

One of the frequent reasons for unsuccessful conception is premature ovarian failure/primary ovarian insufficiency (POF/POI) that is defined as the loss of functional follicles below the age of 40 years. Among the genetic causes the most common one involves the X chromosome, as in Turner syndrome, partial X deletion and X-autosome translocations. Here we report a case of a 27-year-old female patient referred to genetic counselling because of premature ovarian failure. The aim of this case study to perform molecular genetic and cytogenetic analyses in order to identify the exact genetic background of the pathogenic phenotype.

**Results:**

For premature ovarian failure disease diagnostics we performed the Fragile mental retardation 1 gene analysis using Southern blot technique and Repeat Primed PCR in order to identify the relationship between the Fragile mental retardation 1 gene premutation status and the premature ovarion failure disease. At this early onset, the premature ovarian failure affected patient we detected one normal allele of Fragile mental retardation 1 gene and we couldn’t verify the methylated allele, therefore we performed the cytogenetic analyses using G-banding and fluorescent in situ hybridization methods and a high resolution molecular cytogenetic method, the array comparative genomic hybridization technique. For this patient applying the G-banding, we identified a large deletion on the X chromosome at the critical region (ChrX q21.31-q28) which is associated with the premature ovarian failure phenotype. In order to detect the exact breakpoints, we used a special cytogenetic array ISCA plus CGH array and we verified a 67.355 Mb size loss at the critical region which include total 795 genes.

**Conclusions:**

We conclude for this case study that the karyotyping is definitely helpful in the evaluation of premature ovarian failure patients, to identify the non submicroscopic chromosomal rearrangement, and using the array CGH technique we can contribute to the most efficient detection and mapping of exact deletion breakpoints of the deleted Xq region.

## Background

Premature ovarian failure (POF) is an ovarian defect characterized by the premature depletion of ovarion follicles befor the age of 40 years, and its aetiology is still unknown in most cases. Coulon et al. examined 1858 patients with premature ovarian failure, and the age-specific incidence was based on the results: under 40 years of age the incidence was 1:100, and under 30 years of age the incidence was 1:1000 [1]. In addition to a reduction in the duration of fertility, there are other important health issues that may be associated with POF such as an increased risk of overall mortality, cardiovascular diseases, osteoporosis and autoimmune disorders such as diabetes or problems with the thyroid or adrenals [2]. In particular, an association between POF and abnormalities of the X chromosome has been reported several times, and another cause, which can be associated with POF, is the balanced X/autosomal translocations despite their generally neutral clinical effect [3]. In fact, the breakpoints of these aberrations are distributed over the whole X chromosome but in many cases they cluster in a critical region between Xq13 and Xq26 [4-7]. Deletion on the X chromosome reduces both fertility and reproductive lifespan and the basis of studies two loci for Xq-linked POF have been postulated: deletion in POF patients have been localised to chromosome Xq21.3-Xq27 (POF1), while balanced X/autosome translocations have been localised to Xq13.3-Xq21.1 (POF2) [8]. The above-mentioned POF disorder has been attributed to various causes including rearrangements or large deletion of the “critical region” in the long arm of the X chromosome. However, it is interesting that there are several genes which have consistently found to be involved in POF on the X chromosome and autosomes but the POF1 region deletions are by far more commonly associated with POF phenotype. Another abundant reason for the POF is the single gene association that can also be involved in the POF phenotype. Out of the single genes group, the FMR1 gene appears to be the most significant cause of POF disease. The association between FMR1 premutation and POF has been previously investigated at a molecular level by analysing FMR1- related factors such as the repeat tract size. Expansion of (CGG) triplet repeats in the FMR1 gene is associated with several disorders, including fragile X syndrome (FRAXA), fragile X-associated tremor/ataxia syndrome (FXTAS), and fragile X-associated primary ovarian insufficiency (POF). Fragile X syndrome is nearly always characterized by moderate mental retardation in affected males, with full mutation and FXTAS occuring in males who have an FMR1 premutation, and is characterized by late onset progressive cerebellar ataxia and intention tremor. FMR1-related (with the premutation alleles, which may have 55 to 200 CGG) POF (age at cessation of menses <40 years) occurs in approximately 20% of females who have an FMR1 premutation [9]. Since full mutation carriers do not have an increased risk for ovarian dysfunction, the molecular mechanism underlying the association between POF and FMR1 gene premutation alleles, although still unravelled, should not be related to the absence or reduction of the fragile mental retardation protein (FMRP) [10]. The known association between POF and premutation alleles in the FMR1 gene [11] prompted us to initiate fragile X testing. In this case study, we performed a detailed FMR1 gene CGG repeat number assay and cytogenetic analysis such as FISH, G-banding and molecular cytogenetic analysis, which are based on the array CGH technique. The aim of this case study was to perform molecular genetic and cytogenetic analyses in order to identify the exact genetic background of the pathogenic phenotype.

## Case presentation

Genetic testing of the 27 year old female patient was carried out due to suspected premature ovarian failure/primary ovarian insufficiency (POF/POI). Her menses ceased at the age of 25. The first menses (menarche) started at the age of 12. There is no pregnancy or assisted reproductive procedure appearing in her history. Other illnesses, genetic disorders, mental retardation have never occurred in the family. The menopause of her mother occurred at the age of 53. The patient’s body type is average, BMI: 19.4 (average: 18.5 to 24.99). During the examination the disease met the criteria for premature ovarian failure/primary ovarian insufficiency (POF/POI): secondary amenorrhea, ovarian failure before the age of 40, levels of FSH > 40 IU/l in two different measurements and low estrogen levels. The patient has never had surgery significantly affecting both ovaries; ovarian toxic medications have never been used (cytostatic treatment). The patient’s mother has normal karyotype and her father was already deceased. For this case study we presented the details of the molecular and cytogenetic analyses at the index patient which were performed by G-banding; FISH, Southern-blotting, Repeat Primed PCR and array-CGH technique.

## Methods

### Chromosome and fluorescence in situ hybridization analysis

Chromosome analysis was performed on stimulated peripheral blood cultures on metaphase cells with trypsin and Wright Giemsa stain. Fluorescence in Situ Hybridization (FISH) analysis was carried out on methanol/acetic acid-fixed suspensions. Slide preparation for FISH was made according to standard techniques. X, Y centromere specific probes as well as X Painting probe (Cytocell, United Kingdom) were used for evaluation of sex chromosomes and their possible hidden structural abnormalities. Spectrum Green CEP X and Spectrum Red SRY gene specific probe (Abbott, Germany) were used to detect chromosome X copy number and to control the presence of the SRY gene on the chromosome, respectively. Karyotypes and FISH results were described according to the International System for Human Cytogenetic Nomenclature (ISCN 2013).

### Southern blot

Genomic DNA from the patient was isolated from peripheral lymphocytes by the simple salting-out procedure. DNA was subjected to restriction enzyme digestion with *Eco*RI and the methylation sensitive enzyme *Eag*I followed by Southern blot analysis and hybridization using the DNA probe StB12.3 [12]. In unaffected females, two bands are visible: a 2.8 Kb fragment corresponding to the unmethylated X and a 5.2 kb allele representing the methylated X chromosome.

### Repeat primed PCR

The exact number of CGG repeats was determined by Repeat Primed PCR technology (Amplidex, Asuragene). The genomic DNA sample was diluted (20 ng/μl), and then for PCR was used 2 μl. The PCR reaction was carried out in three primers: the FMR1 gene-specific primers (forward and reverse, FAM-labeled) and a CGG repeat specific primer. The PCR products were separated by size, using capillary electrophoresis. The statistical analyzes were performed with FMR1AnalysisMacro_version 2.1.1 software and GeneMapper software.

### Array CGH analysis

Array CGH analysis was performed according to the manufacturer’s protocol on genomic DNA ISCA plus design array of Nimblegen Roche containing 1.4 M probes per sub array. This microarray provides a mean average resolution of approximately 15–20 Kb on chromosomes to detect chromosomal imbalances throughout the whole genome. The CGH protocol involves independent labelling of the patient (test DNA) and the reference genomic DNA (Human Genomic DNA, Promega Madison, WI U.S.A.) with Cy3 and Cy5 dyes using a NimbleGen Dual-Color DNA Labelling Kit (Roche NimbleGen Inc.). Cohybridization of these DNAs to a NimbleGen CGH arrays were performed for 72 hrs at 42˚C. Following hybridisation, the array was washed and dried at room temperature using the wash buffer kit (Roche NimbleGen Inc.). Array CGH was scanned on NimbleGen MS 200 microarray scanner and data was extracted and analysed using NimbleScan software and SignalMap and Deva 1.1 software (Roche NimbleGen Inc.). DNA CNVs were mentioned as gain or loss as a linear ration, and the length of the variation was given in megabase (Mb).

## Results

**Figure 1:**
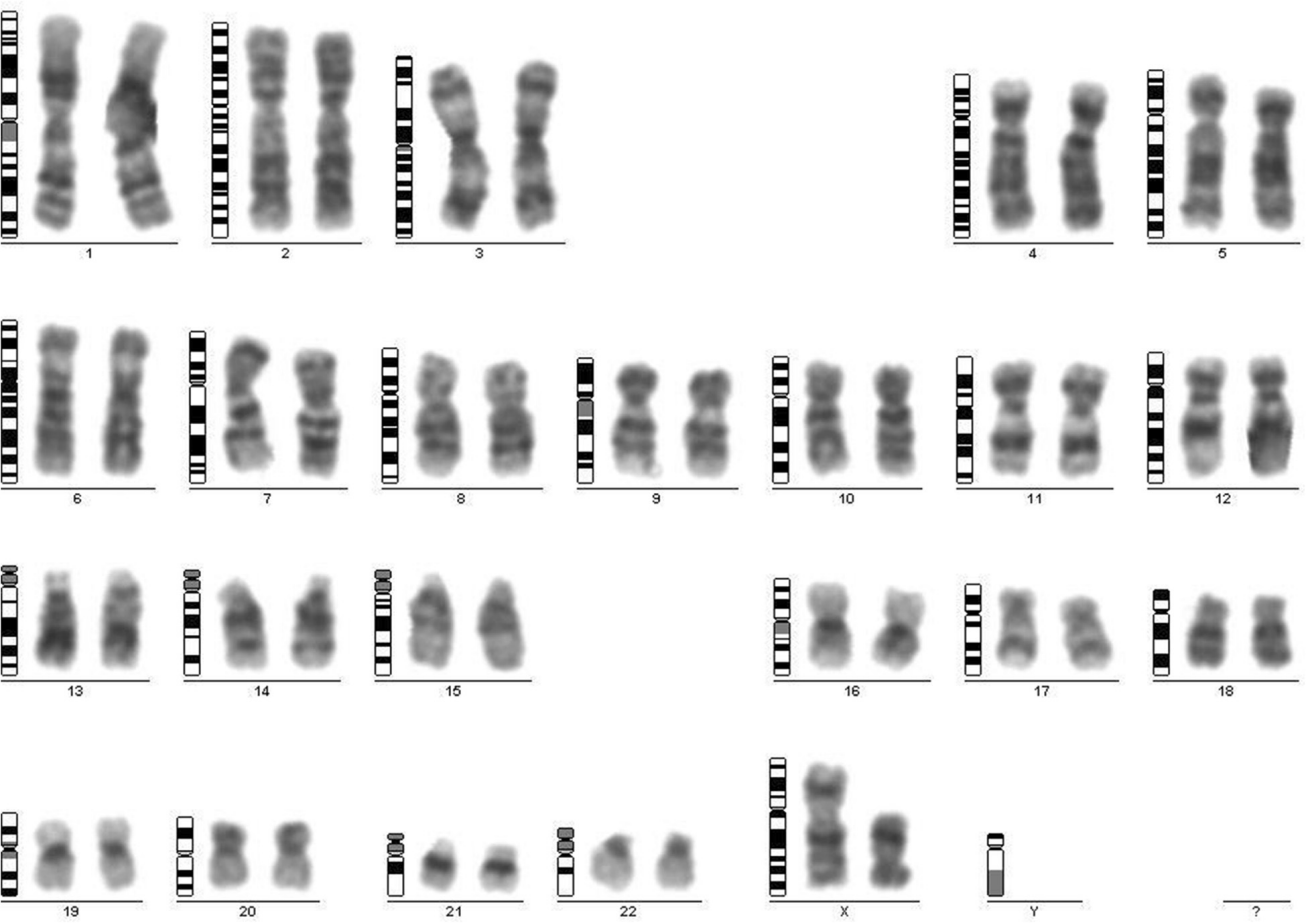
**G-banding analysis.** The karyotype of the patient with Xq21-q28 deletion of the dominant cell line.

**Figure 2:**
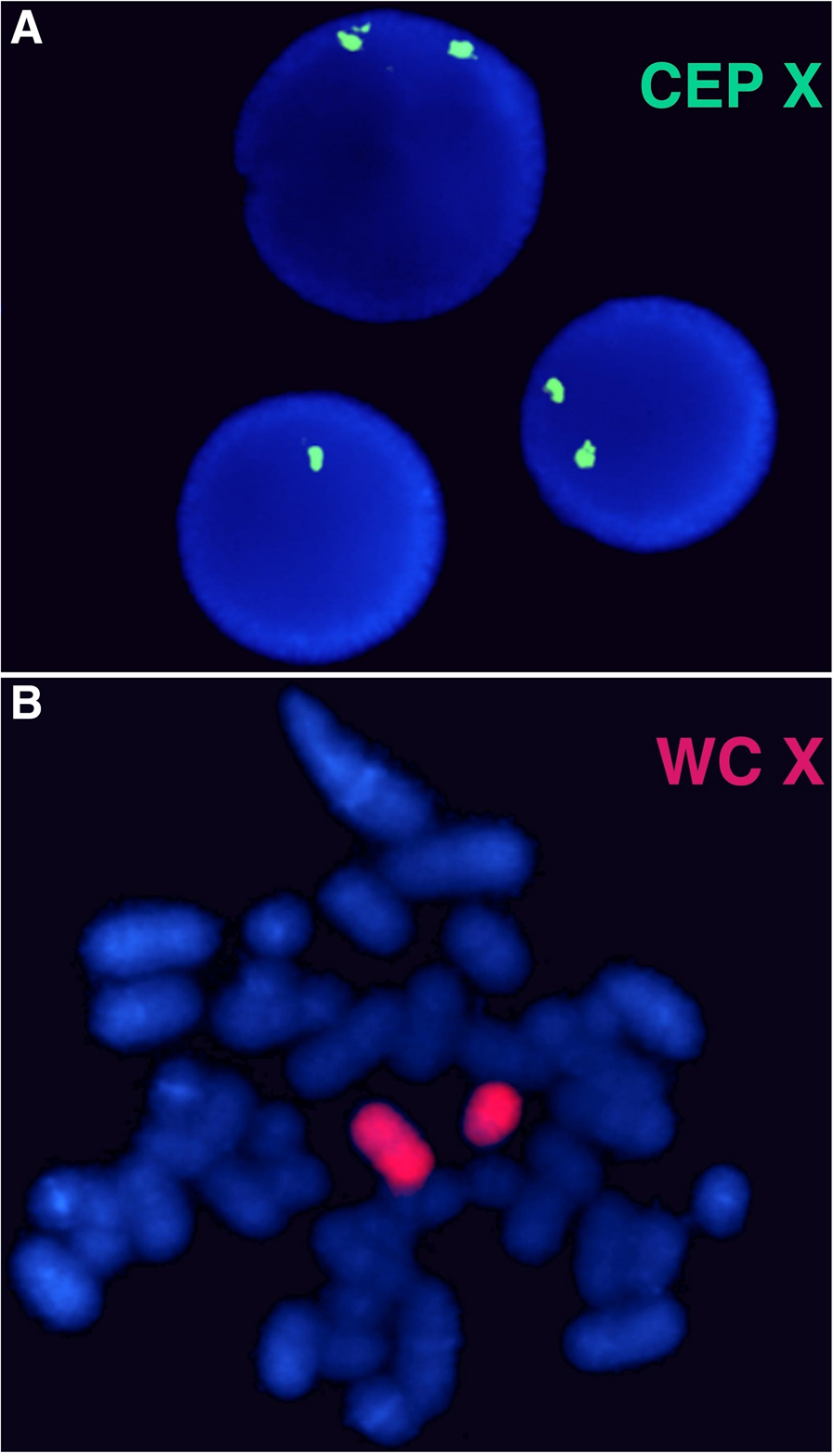
**FISH analysis.** For FISH analysis using chromosome X centromere specific probe (CEP X) which shows normal female pattern (two green signals) in 90% of cells and X monosomy (one green signal) in 10% of interphase cells **(a)**. The whole painting chromosome X (WC X) identified a normal and a smaller size red colored X chromosome and excluded the possible X chromosome translocation **(b)**.

**Figure 3:**
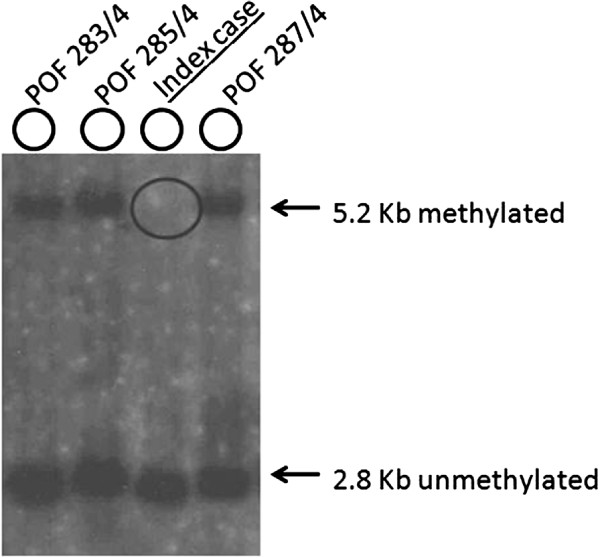
**Picture of Southern blot analysis.** EcoRI and EagI double digested DNA samples using radioactive-labelled Stb12.3 probe for Southern blot hybridization. Arrows indicated the 2.8 Kb unmethylated and the 5.2 Kb methylated fragments size. For the case sample we can define the missing 5.2 Kb methylated fragment (circle compassed). POF 283/4, POF 285/4 and POF 287/4 cases indicated those female samples who did not carry FMR1 gene premutation and the background of the POF phenotype should be withstand other genetic deviation. For all these cases we identified two X chromosome normal allels (2,8 kb unmethylated and 5.2 kb methylated).

**Figure 4:**
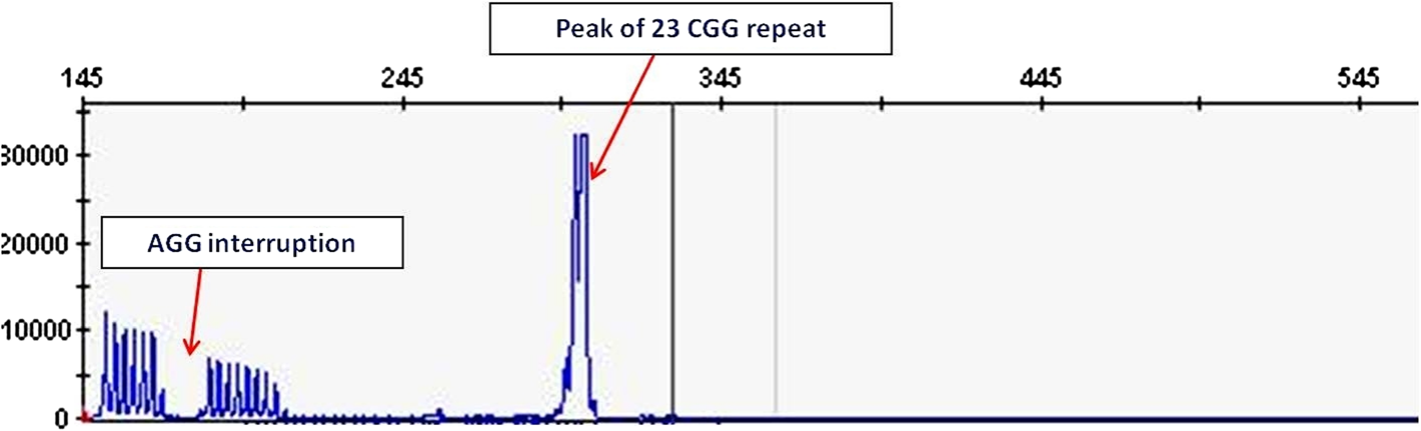
**Picture of repeat primed PCR analysis.** Repeat-primed PCR analysis revealed a peak, which corresponds to a 23-CGG with only one AGG interruption.

**Figure 5:**
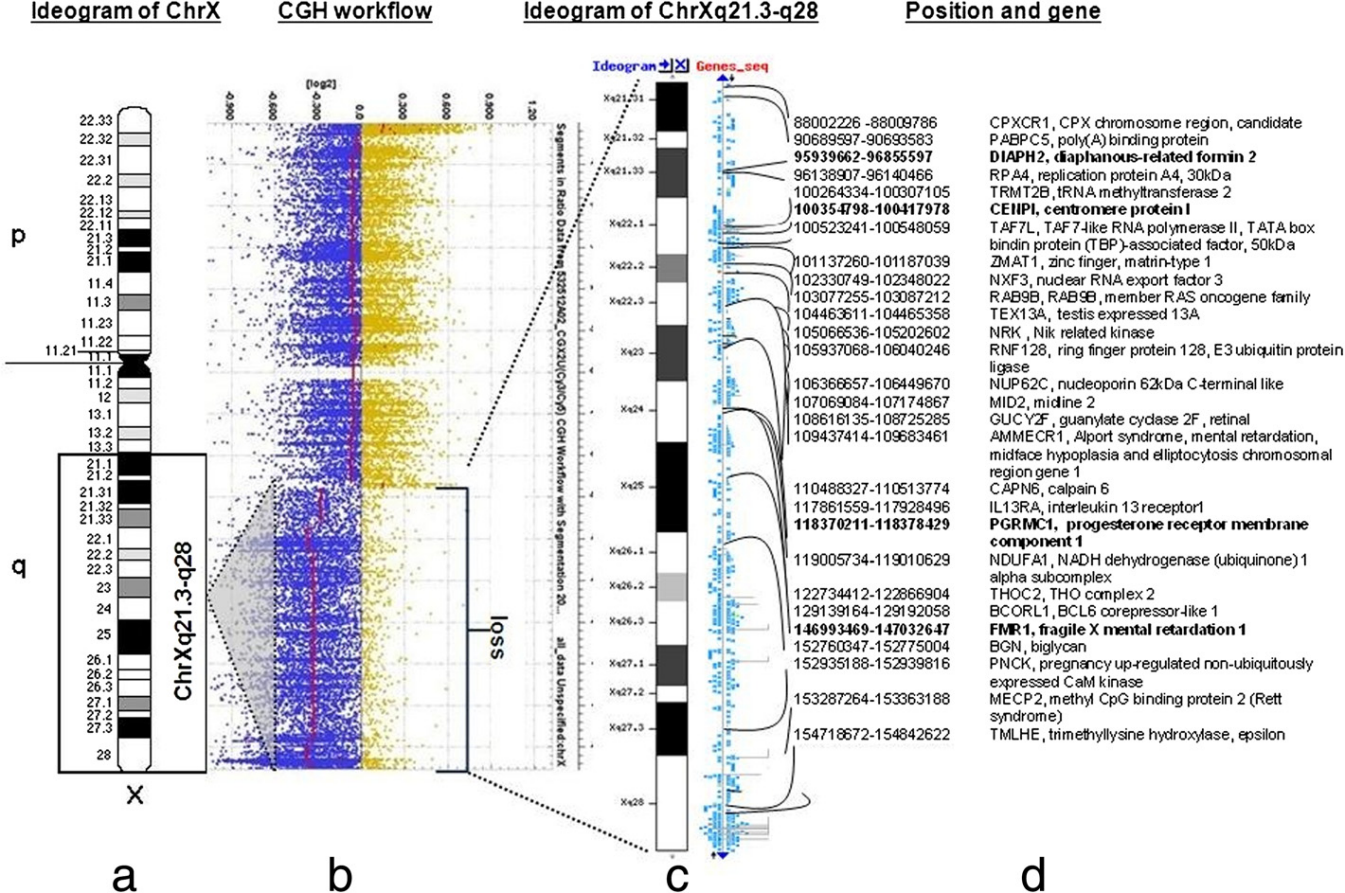
**NimbleGen ISCA plus CGX design profile for X chromosome. a.)** The ideogram (below: black, grey and white bars) delineates genomic regions with the cytogenetic bands on the X chromosome. An 67.355 Mb sized loss on chromosome Xq21.3-q28 for the female patient and a black rectangle indicate the length of the loss. **b.)** Array-CGH workflow. The CGX ISCA plus array showed a 67.355-Mb loss which presented one copy. The 67.355 Mb deleted chromosome segment (GRCh37/hg19; ChrX: 87842016–155255380) is denoted by a bar red line below zero. The blue and yellow dots depict the normalized ration on every probe on the X chromosome. **c.)** Schematic representation of the chrXq21.3-q28 enlarged region. **d.)** Next the ideogram listed genes and positions which are affected this patient. This affected region contains 1818 genes and we visualized some of them. We signed with bold font those genes, which can x play a roll to induce the POF/POI phenotype.

For the POF/POI affected patient, the G-banded analysis based on 30 metaphases revealed two cell lines, the argest has a structural (Xq deletion) and the smallest a numerical (X monosomy) chromosome abnormality. The mosaic karyotype was the following: 46,XX,del(X)(q21q28)[25]/45,X[5] (Figure 1). The patient’s mother has normal karyotype and as the father was already deceased. This deletion in a male would be incompatible with life and so we can stated that the deletion is “de novo”. The FISH examination with the X centromere*/SRY* specific probe-based on 200 interphase cells-detected two X chromosomes in 90% of cells and X monosomy in 10% of cells and no *SRY* signals respectively (Figure 2a). The whole painting chromosome X FISH probe did not disclose X chromosome balanced translocation and identified a normal and a smaller size X chromosome in 88% and one normal size X chromosome in 12% of cells (Figure 2b). For southern blot in this case we detected one FMR1 allele of X chromosome which was the 2.8 Kb size and unmethylated, and the 5.2 Kb methylated allele was not detected (Figure 3). For southern blot analysis for the index patient we can detect only the active X chromosome so this is why we had to make the Repeat-primed PCR in order to identify the CGG number and the exact allele number. Repeat-primed PCR analysis revealed a peak, which corresponds to a 23-CGG, and we can detect only one FMR1 gene allele. The method is also suitable for detection of AGG sequences interrupting CGG repeats. The AGG repeats stabilize the CGG repeats containing sequences. The more the number of AGG interruptions, the less likely it is to grow in the next generation of the number of CGG repeats. At the index patient we determined only one AGG interruption (Figure 4). Regarding the result of the cytogentics analysis we identified a large deletion on the X chromosome (measure: 67.355 Mb) and in order to identify the exact breakpoints, we made the array CGH technique and we defined an X chromosome loss that is located at ChrX:87842016–155255380 (ChrX q21.31-q28) based on the Human genome GRCh37/hg19 assembly (Figure 5).

## Discussion

A 27 year old woman with premature ovarian failure including ceased menses at the age of 25 and elevated FSH? 40 mIU/ml and low estrogen levels. She carried a 67.355 Mb deletion on one of the X chromosomes and the exact breakpoints boundaries were identified by an oligonucleotide aCGH analysis. In this region, in total 795 genes were located and up until now, ten genes (POF1B; BHLHB9; DACH2; DIAPH2, FMR1; FMR2; XPNPEP2; PGRMC1, CENP1, BCORL1) have been identified as the ones associated with POF (Table 1). The frequencies of these genes in POF are different, the highest value is 3-15% at the FMR1 gene premutation cases [13,14] and followed PGRMC1 variants with 1–5% prevalence [15] and for the other six genes the frequencies are still unknown in POF/POI disease. The other genetic aspect which can effect the POF/POI phenotype is the chromosomal anomalies. These aberrations can be liable for the POF/POI phenotype in 8.8-33% of women [16] and 10-15% of the cases are X chromosome abnormalities, such as numerical and structural aberrations (deletions, inversions and X/autosome translocations) [17,18]. Theoretically, fertility impairment in patients with chromosomal abnormalities can be explained in various ways. First of all, chromosomal anomalies might disrupt a gene that is important for gonadal function [19] and structural rearrangements involving the X chromosome may disrupt the normal pairing at meiotic arrest [3]. Focus on the candidate genes that may be implicated in the POF phenotype, the first gene that we must exam, is the POF1B gene. The function of this gene is still unknown, although it binds to actin and has some homology to myosin heavy chain, so it has a remarkable role in the chromosome pairing procedure. This gene product is expressed in the ovaries during early embryonic development [20] and the mutation of the POF1B gene affects the actin binding action that may be obligate at meiotic chromosome pairing and apoptosis [21]. Deletion of the other candidate gene the BCORL1 may lead to insufficient repression of apoptosis resulting in atresia of ovarian follicles. For the chromosome X there is another candidate gene (CENP1) that has been shown to have a critical role in chromosome segregation and the deletion of this gene may cause cell death. It has also been suggested that these structural abnormalities may exert an epigenetic effect influencing the expression of X-linked or autosomal ovary-expressed genes [22]. As the molecular study revealed, the BCORL1 gene has a repressor activity through an association with histone deacetylase, suggesting that they are involved in its function as a corepressor. Disposing the histone associated epigenetic system; the BCORL1 gene may play a role in the epigenetic modification but as scientific literature suggests, genes interruption is not the major cause of pathological phenotype. In particular the breakpoint in the X-autosome translocation in the POF2 interval falls outside gene coding regions, and it has been suggested that the observed effect on expression of ovary and oocyte autosomal and X-linked genes flanking the translocation breakpoints may arise as a consequence of long range effects on promoter activity [22]. Second, some of these genes can influence hormone levels and tissue response to hormones that may impact on efficient oocyte development and maturation. For example, in addition to a role in centromere formation, the CENP1 gene is involved in gonadal tissue response to FSH [23].

**Table 1:** POF associated genes at the critical region on chromosome X which are affected at patient

**Gene acronym**	**Gene name**	**Chromosome localisation**	**OMIM**	**Phenotype**	**Protein**
POF1B	Premature ovarian failure1B	ChrXq21.1-q21.2	300603	Premature ovarian failure 2B	This gene is expressed at trace levels in mouse prenatal ovary and is barely detectable or absent from adult ovary, in human and in the mouse respectively.
BHLHB9	Basic helix-loop-helix domain-containing class B 9	ChrX q21.1		Premature ovarian failure	Other members of this gene family encode proteins which function as transcription factors, either enhancing or inhibiting transcription depending on the activity of the DNA binding proteins.
DACH2	Drosophila dashsund	ChrX q21.2	300608	Premature ovarian failure	This gene is one of two genes which encode a protein similar to the Drosophila protein dachshund, a transcription factor involved in cell fate determination in the eye, limb and genital disc of the fly.
DIAPH2	Homologue drosophila	ChrXq21.33	300108	Premature ovarian failure	The product of this gene belongs to the diaphanous subfamily of the formin homology family of proteins. This gene may play a role in the development and normal function of the ovaries.
CENPI	Centromeric protein 1	ChrXq22.1	300435	Involved in the gonadal tissue response to FSh and assembly of the kinetochore	It has a critical role in chromosome segregation and with deletions candidate for human X-lined disorders of gonadal development and gametogenesis.
PGRMC1	Progesteronereceptor memebrane component-1	ChrXq24	300435	Premature ovarian failure	This gene encodes a putative membrane-associated progesterone steroid receptor. The protein is expressed predominatly in the liver and kidney.
BCORL 1	BCL6 Corepressor-like 1	ChrXq25-q26.1	300686	BCORL1 interacted with class II histon deacetylases suggesting that they are involved in its function as a corepressor	Deletion of BCORL 1 gene may potentially lead to insufficient repressor of apoptosis resulting in atresia of ovarian follicles.
XPNPEP2	Propyl aminopeptidase	ChrXq26.1	300145	Angioedema induced by ACE inhibitors, susceptibility to	XPNPEP2, the X-linked gene that encodes membranous aminopeptidase P (APP), has been reported to associate with APP activity. The membrane aminopeptidase P (XPNPEP2) is largely limited in distribution to endothelia and brush border epithelia.
FMR1	Fragile X mental retardatin 1	ChrXq27.3	309550	Fragile X syndrome Fragile X tremore/ataxia syndrome Premature ovarian failure 1	The protein encoded by this gene binds RNA and is associated with polysomes. The encoded protein may be involved in mRNA trafficking from the nucleus to the cytoplasm.
FMR2/AFF2	Fragile X mental retardation 2	ChrXq28	300806	Mentar retardation, X-linked, FRAXE type	This gene encodes a putative transcriptional activator that is a member of the AF4\FMR2 gene family.

For the index patient as we mentioned before using the Southern blot assay, we detected a normal and active X chromosome and the absence of the methylated FMR1 allele, according to Lyon hypotheses, should be inactivated in 50% of cells [24]. As scientific studies described, the haploinsuffiency of the genes, which are located in the missing region on one of the chromosome X, could be a promising explanation for the POF disease background, especially when it involves Xq28. A haploinsufficient gene is described as needing both alleles to be functional in order to express the wild type. The lack of expression of those missing genes that normally escape X inactivation may threaten ovarian function [25]. Based on the scientific studies we can conclude that with this POF, the affected patient may be configured by abnormal X chromosome pairing and the epigenetic modification [26]. The epigenetic effects would be supportable if the patient had a POF affected relative carrying the same deletion. In this case we confirmed a “de novo” X-chromosome deletion, so we cannot make any phenotype comparison. We conclude that cytogenetic analysis might be the first step in the investigation of POF/POI, as it might make some subsequent analysis steps unneccessary (i.e. Southern blot and PCR).

## Conclusion

Here we present a patient affected with POF disease, where molecular and cytogenetic analyses revealed that she was a carrier of a large deletion spanning from Xq21.31-q28. Sample DNA of the POF phenotype affected women were collected so as to identify the exact molecular genetics background. As the scientific studies suggested, the most frequent gene mutation that can cause the POF phenotype, is the CGG triplet repeat number increasing which denoted that these females are the permutation status carrier. For the index patient we confirmed a large deletion on one of the X chromosomes and we couldn’t detect premutation status at the FMR1 gene. In order to identify the exact breakpoints of the X chromosome deletion, we made the array CGH analyses. As we verified the deletion on the database, we realised that this deletion region consisted of 795 genes, and 10 of them were considered as POF associated genes. Comparing the phenotype and the molecular genetic results, we concluded that for this patient, we established the POF1 disease and offering the prenatal diagnostic at possible future pregnancies is crucial.

## Consent

Written informed consent was obtained from the patient for publication of this case report and accompanying images.

## Abbreviations

BCORL1: BCL 6 corepressor-like-1; BHLHB9: Basic helix-loop-helix domain-containing class B; CENP1: Centromeric protein 1; CGG: Citosine-guanine-guanine trinucleotide; CGH: Comparative genomic hybridisation; CNV: Copy number variation; DACH2: Drosophila dashsund homolog 2; DIAPH2: Drosophila diaphanous homolog 2; FISH: Fluorescens in situ hybridisation; FMR1: Fragile mental retardation 1 gene; FMR2: Fragile X mental retardation 2; FMRP: Fragile mental retardation protein; FRAXA: Fragile X syndrome; FSH: Follicle stimulating hormone; FXTAS: Fragile X-associated themor ataxia syndrome; PGRMC1: Progesterone receptor membrane component-1; POF: Premature ovarian failure; POF1B: Premature ovarian failure 1B; XPNEP2: Propyl aminopeptidase gene.

## Competing interest

The authors declare that they have no competing interests.

## Authors’ contributions

AB cared for the patient. AB and HP contributed to data collection and the first draft of the manuscript. HP and VK performed the array CGH analysis and Southern blot examination, HP and AB performed Repeat Primed PCR, IH and GF performed FISH examination, JC and AM performed cytogenetic examination. JJR, VK read and approved the final manuscript. All authors read and approved the final manuscript.
